# The extent of wind-mediated dispersal of small metazoans, focusing nematodes

**DOI:** 10.1038/s41598-018-24747-8

**Published:** 2018-05-01

**Authors:** Christoph Ptatscheck, Birgit Gansfort, Walter Traunspurger

**Affiliations:** Animal Ecology, Bielefeld University, Konsequenz 45, 33615 Bielefeld, Germany

## Abstract

Wind-mediated transport is an important mechanism in the dispersal of small metazoans. Yet, concrete dispersal rates have hardly been examined. Here we present the results of an one-year field experiment investigating the composition and dispersal rates of aeroplankton. To gain insights into the dynamics of dispersal at the species level, we focused on nematodes, worldwide the most common metazoan taxon. Among the six taxa collected in this study (nematodes, rotifers, collembolans, tardigrades, mites, and thrips), nematodes had the highest dispersal rates (up to >3000 individuals m^−2^ in 4 weeks, 27 species identified) and represented >44% of aeroplankton. Only living nematodes, and no propagules, were dispersed. All taxa had a higher dispersal potential in environments linked to the source habitat, evidenced by the much higher deposition of organisms in funnels placed on the ground than on the rooftop of a ten-story building. Nematodes under conditions of high humidity and wind speed had the highest dispersal rates, while increasing temperatures and dryness had a significantly positive impact on the wind drift of mites and thrips. The results indicated that wind dispersal over long distances is possible. The notable organismal input by wind dispersal may contribute to biodiversity and ecosystem functions.

## Introduction

Dispersal is a vital component of an organism’s life-history^1^, and the potential for dispersal determines the distribution, abundance, and thus, the community dynamics of species at different sites^2–4^. A new habitat must first be reached before filters such as organismal abilities and adaptations, the quality of a habitat, and the established biocoenosis determine the colonization efficiency of a species^5^. While larger animals can cover distances on their own and actively seek suitable habitats, small (<2 mm) organisms are often passively dispersed^5^, resulting in their more ubiquitous occurrence^6^. While active dispersal accounts for rather predictable distribution patterns, passive dispersal leads to a more randomized immigration of organisms^2^. Mechanisms for passive dispersal are the transport on (epizoochory) or in (endozoochory) larger animals (e.g., flying insects, birds, or mammals) and the erosion by wind^5^.

Often cited as important requirement for effective wind dispersal is the presence of propagules (e.g., resting eggs, cysts, ephippia, juvenile and adult resting stages)^5,7,8^, which also enables organisms to survive unfavorable environmental conditions until they enter a suitable habitat. These dispersal units can be blown from surfaces such as soil, moss, and the desiccated sediments of temporal waters. The passively dispersed organisms are typically pioneer colonizers^9–11^. However, wind-drifted species vary in their vagility (probability to be transported with the wind)^12^, with the weight and form of the propagules, and therefore, the wind speed required for their transport^13^, determining the dispersal distance. For example, in nematodes resting eggs are less effectively transported by wind than other life stages^14^, while organisms in anhydrobiosis are lighter and thus more readily transported than hydrated forms^15,16^. Because different organisms are, for the most part, not dispersed over the same distances, source habitats are also important, with the number of organisms contained in air declining with increasing distance from the source system^9,17^. The distances covered by small metazoans range from a few meters^17^, to several hundred meters^9^, and up to several kilometers^14^. While the wind dispersal of aquatic organisms is possible even during the wet phase of a transiently aquatic habitat^5^, during the dry stages a larger number of dormant propagules are exposed to wind and thus dispersed^8,17,18^. Freshwater organisms that must “cross the dry ocean”^5^ to enter new aquatic island systems will be passively dispersed more successfully than terrestrial taxa^5^. However, numerous taxa from both soil and freshwater systems have been captured from the air (e.g., bacteria, several algae, ciliates, flagellates, rotifers, crustaceans, mites, and tardigrades)^9,17–19^. While these have been qualitatively well studied, accurate estimates of their dispersal rates are lacking.

Few investigations of the aeroplankton specifically mention nematodes^8,9,17^, the most common metazoan taxon and an essential trophic link between unicellular organisms (e.g., bacteria) and larger organisms (e.g., tardigrades, copepods, flatworms, and fishes)^20–23^. For nematodes, anhydrobiosis is a widespread strategy allowing them to survive unfavorable conditions for months and even years^24–26^. Accordingly, nematodes can be readily dispersed by wind. However, as reported by Vanschoenwinkel *et al*.^17^, nematodes account for only ~1% of the wind-drifted metazoans. Among the habitats colonized by nematodes are those that are strongly exposed to wind erosion as e.g., littorals of permanent waters, soils, mosses, dead wood, and tree bark^27–30^. In addition, temporal waters, such as phytotelmata, were shown to be colonized by numerous nematode species already within a few days^11,31^.

The main goals of our study were to investigate the dispersal potential of small metazoan taxa (<2 mm), especially nematodes, their dispersal rate, and the impact of meteorological parameters on wind dispersal. These questions were addressed in an intensive one-year experimental field study documenting the seasonality of wind dispersal at two selected locations. A suitable experimental design for the determination of dispersal potential is the use of new, artificial habitats (e.g., artificial water bodies) that can be newly colonized^5^. Because of priority effects and other biotic and abiotic interactions, organisms entering a habitat can easily disappear^5^. Thus, for an effective measurement of the dispersal rate, incoming organisms should be captured directly from the air, before they are disrupted by local regulators^9,17^. For this reason, we conducted our study using both vessels filled with formaldehyde to collect wind-drifted organisms and vessels filled with water to document the viability of nematode propagules.

We expected that metazoan taxa, and especially nematodes, due to their frequent occurrence in wind affected habitats, are an important component of the aeroplankton (hypothesis H1). We also predicted that more organisms will be transported by wind at a location with close distance to potential immigration sources than at a location where the distance from those source habitats is larger (hypothesis H2.1) and that, for nematodes, a higher number of species would occur in this location with higher proximity to immigration sources (hypothesis H2.2). A further hypothesis (H3) was that the rate and extent of wind dispersal is affected by meteorological factors; thus, because drought is a crucial factor for wind dispersal, low humidity and high wind speeds will support organismal drift.

Focusing on nematodes, we documented their species composition, size classes, and sex distribution, anticipating that mainly nematodes with a short body length (<0.75 mm) would be able to reach habitats with less proximity to source habitats (hypothesis H4). Finally, as already shown for several other taxa, we predicted that (H5) nematodes are dispersed mainly as propagules (anhydrobiotic stages).

## Material and Methods

### Experimental setup

**Figure 1:**
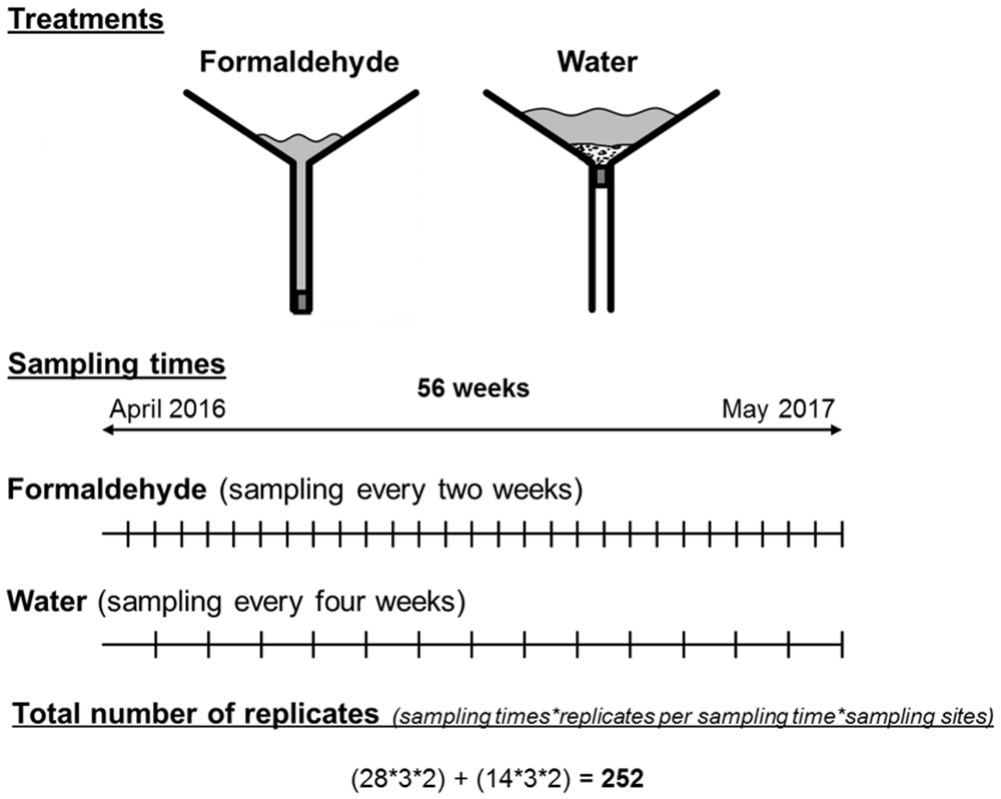
Experimental setup of the two investigated treatments. One set of funnels was filled with formaldehyde and sampled every 2 weeks. A second set of funnels was filled with water and sediment and sampled and refilled after 4 weeks. Both the formaldehyde- and water-filled funnels were placed in a natural environment (meadow) and on the rooftop of a building at Bielefeld University.

Plastic funnels with an opening of 20 cm in diameter were used to collect wind-dispersed organisms. Based on our initial hypotheses, two different treatments were implemented. For the first, the tube of each funnel was plugged at the bottom and the funnels then filled with 60 ml of 37% formaldehyde (Fig. 1). The funnels were also marked to indicate the liquid level when the formaldehyde was diluted to 4% by rain. For the second, the funnel tubes were plugged at the top and the funnels then filled with 60 g of sand (0.4–1.4 mm grain size; previously autoclaved at 121 °C for 20 min) and 500 ml of tap water to create a refuge for incoming aquatic organisms. Based on the results of preliminary investigation (unpublished data) we could exclude the presents of metazoan within the water.

One experimental setup (ground) was placed on a meadow at a distance of 8 m from a stand of trees (*Acer platanoides*, *Carpinus betulus*, *Fagus sylvatica*, and *Quercus robur*) located at Bielefeld University (Germany). The stand is surrounded by several ponds and natural vegetation units (e.g., hedges and old trees). The second experimental setup (roof) was placed on the roof of a Bielefeld University building (10^th^ floor, 35 m above the ground). The roof is covered by a thin layer of stones (4–9 cm) to hinder the accumulation of organic particles and plants. The two study locations were located 200 m apart. The funnels of the two treatments (water and formaldehyde) were separated at a distance of 1–3 m from each other, with replicates (n = 3 per treatment) separated by 5–8 cm. The upper opening of the funnels was located 40 cm above the ground.

### Sampling

The duration of the field trial was 56 weeks, beginning in April 2016 (Fig. 1). The formaldehyde-filled funnels were emptied every 2 weeks, and the water-filled funnels every 4 weeks. However, after strong precipitation, when the formaldehyde was diluted down to a concentration of <4%, the funnels filled with formaldehyde were emptied between sampling dates. In this case, the samplings of a 2-week interval were pooled. Conversely, after a long period of desiccation, the formaldehyde was diluted with water. In the water treatments, an overflow was not prevented, but water was added when the water in the funnels had nearly desiccated. This was necessary to enable the survival of the organisms and to avoid cross contamination between neighbored funnels. Especially the shape of the funnels makes the drift of organisms between the funnels of the same location unlikely.

At the sampling dates, the funnels of both treatments were thoroughly rinsed with water and the respective suspensions sieved through 5-µm meshes to retain the accumulated organisms. To prepare the emptied funnels for the next interval, new formaldehyde or a sediment-water mixture was added.

In addition, to qualitatively assess the nematodes that had colonized the roof of the university building, stones, organic material and plants were collected every 2 weeks (control).

All samples were fixed in 37% formaldehyde (final concentration >4%) and stained with Rose Bengal.

### Counting and classification of organisms

The abundances of the dispersed metazoans were evaluated using a LEICA L2 stereomicroscope (40× magnification). The nematodes were assigned to specific size classes (<0.25 mm, 0.25–<0.5 mm, 0.5–<0.75 mm, 0.75–<1 mm, 1–<1.25 mm, 1.25–1.5 mm). Each sample contained a maximum of 50 nematodes, prepared as described by Seinhorst^32^. The nematodes were identified to the species level (Leica Dialux microscopy observations, 1250× magnification). The dispersal rates [individuals (Ind.) m^−2^ accumulated in 4 weeks] were calculated based on the results of the formaldehyde treatments (extrapolated from an area of 314.2 cm^2^).

### Meteorological impact

Meteorological data were obtained from a meteorological station located in Bielefeld-Deppendorf (Germany). The relevant parameters were: mean temperature (°C), mean wind speed (km h^−1^), mean precipitation (mm^−1^), and mean humidity (%).

A generalized linear model (GLM) was applied to test whether the number of counted taxa in the formaldehyde funnels (all replicates per sampling date and the counts from the roof and ground were pooled) was influenced by these parameters. In this model, all predictors were treated as continuous variables. The GLM was based on a negative binomial distribution, as is typically done for count data^33^.

Since the ratio of the sample size and number of estimated parameters included in the models was <40, a second-order Akaike information criterion (AIC_c_) was used to evaluate the models^34^. A forward-selection procedure using likelihood ratio tests for nested models was performed to determine the significances of the individual predictors in the model identified using χ^2^ tests^35^.

The GLMs were calculated using the ‘*glm*.*nb*’ function of the ‘*MASS*’ package^36^ in the R environment^37^.

### Data Availability

The datasets generated during and/or analysed during the current study are available from the corresponding author on reasonable request.

## Results

### Collected organisms

**Figure 2:**
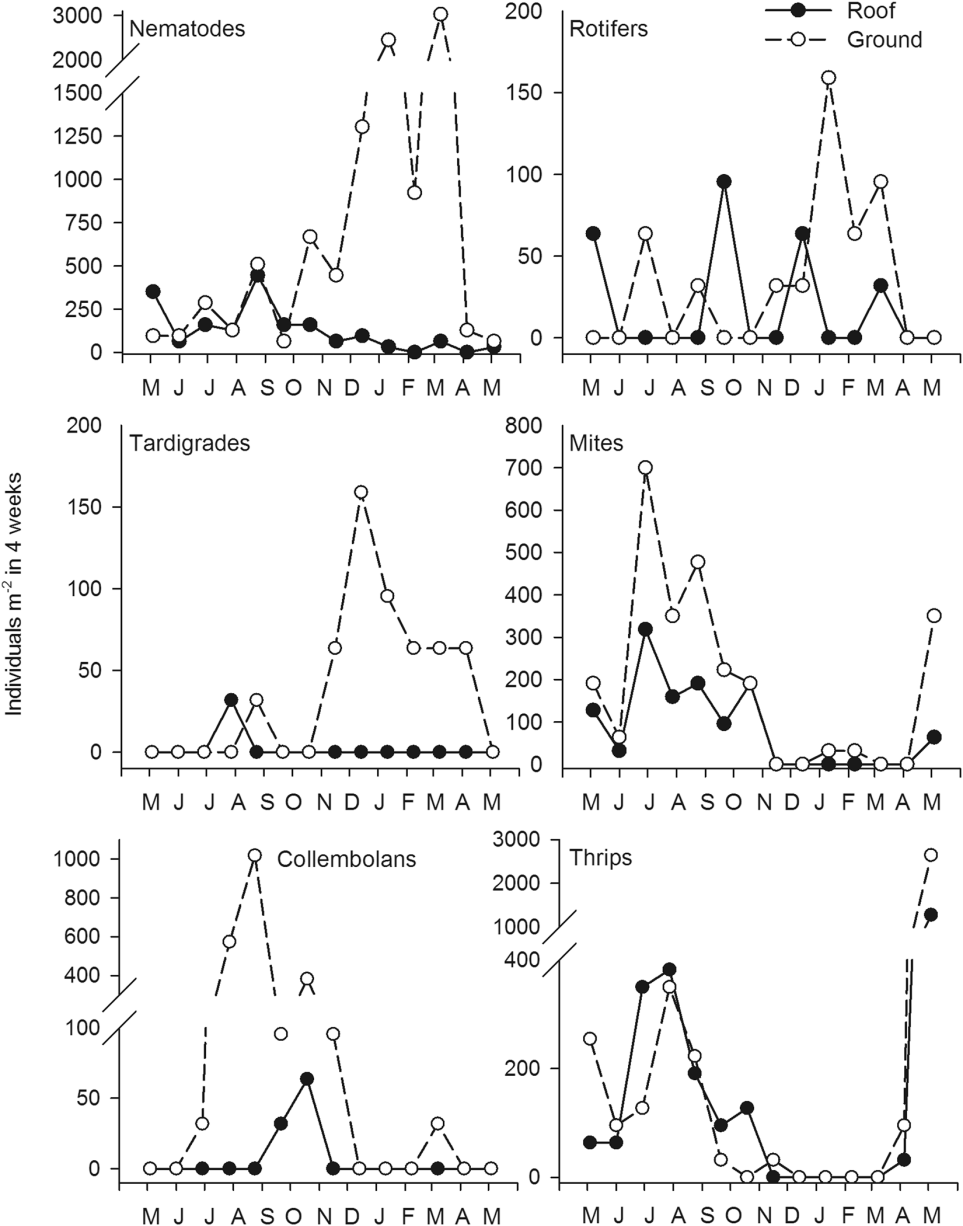
Dispersal rates (Ind. m^−2^ in 4 weeks) of the wind-drifted taxa (nematodes, rotifers, tardigrades, mites, collembolans, and thrips), collected on the ground (filled circles) and on the roof (blank circles). Each data point represents the summed organism number of three replicates subsequently extrapolated to 1 m^2^.

In the formaldehyde treatments, 617 organisms belonging to six wind-dispersed taxa [nematodes, rotifers (mainly Bdelloidea and single *Lecane* species), tardigrades, mites, collembolans and thrips] were present in the funnels on the ground and 136 in those on the roof (Table 1). Numerous adult insects, especially dipterans and hymenopterans, were also detected but were not included in the aeroplankton. All counted organisms were tainted by rose Bengal, and therefore are contamination by metazoan during the counting process could be excluded. Nematodes and thrips made up the major part of the wind-drifted organisms (44.7% and 31.3% on the ground; 46.3% and 18.7% on the roof, respectively). Nematodes were detected in samples from 92.9% of the sampling dates, followed by mites and thrips (each 64.3%) (Fig. 2). Based on the formaldehyde treatments and a 4-weekly input, the highest dispersal rates at both sampling locations were calculated for nematodes: 3021 Ind. m^−2^ in 4 weeks on the ground; 445 Ind. m^−2^ in 4 weeks on the roof (Table 1).

**Table 1:** Mean number of individuals (Ind.) m^−2^ collected within 4 weeks (N = 14, 14 months of sampling) from funnels filled with formaldehyde (±SD), the mean percentage of the aeroplankton (N = 14), and the maximal dispersal rate (Ind. m^−2^ in 4 weeks) from the ground and roof treatments.

		Ground	Roof
Individuals found		N = 617	N = 136
Nematodes	Mean no. Ind.	726.8 (933.1)	124.9 (129.3)
	Mean percentage	44.7	46.3
	Max dispersal rate (Ind. m^2^ in 4 weeks)	3021	445
Rotifers	Mean no. Ind.	34.1 (47.3)	18.2 (32.2)
	Mean percentage	8.0	2.0
	Max dispersal rate (Ind. m^2^ in 4 weeks)	95	64
Tardigrades	Mean no. Ind.	38.6 (48.6)	2.3 (8.5)
	Mean percentage	0.3	3.9
	Max dispersal rate (Ind. m^2^ in 4 weeks)	64	32
Mites	Mean no. Ind.	84.0 (100.3)	186.3 (215.6)
	Mean percentage	14.3	17.8
	Max dispersal rate (Ind. m^2^ in 4 weeks)	191	127
Collembolans	Mean no. Ind.	159.0 (300.6)	6.8 (18.4)
	Mean percentage	1.4	11.2
	Max. dispersal rate (Ind. m^2^ in 4 weeks)	382	64
Thrips	Mean no. Ind.	274.8 (933.1)	184.0 (337.7)
	Mean percentage	31.3	18.7
	Max. dispersal rate (Ind. m^2^ in 4 weeks)	636	350

The highest nematode dispersal rates in the formaldehyde treatments were measured on the ground between December 2016 and March 2017 (76% of the collected nematodes) and on the roof between May 2016 and November 2016 (87.2%) (Fig. 2). The tardigrades showed a similar seasonal progression but lower dispersal rates (up to 636 m^−2^ in 4 weeks). For the collected arthropods (mites, collembolans, and thrips) the highest dispersal rates also were measured between May 2016 and November 2016, and at both sampling locations. For rotifers, there was no clear trend. In July 2016, the nematode dispersal rates on the ground were equal to those on the roof but in May and September 2016 they were lower. At the other sampling dates, the dispersal rates on the roof were between 12.5% and 100% lower. All sampled nematodes were living individuals, with no indications of propagules or anhydrobiotic stages.

### Nematode composition

**Figure 3:**
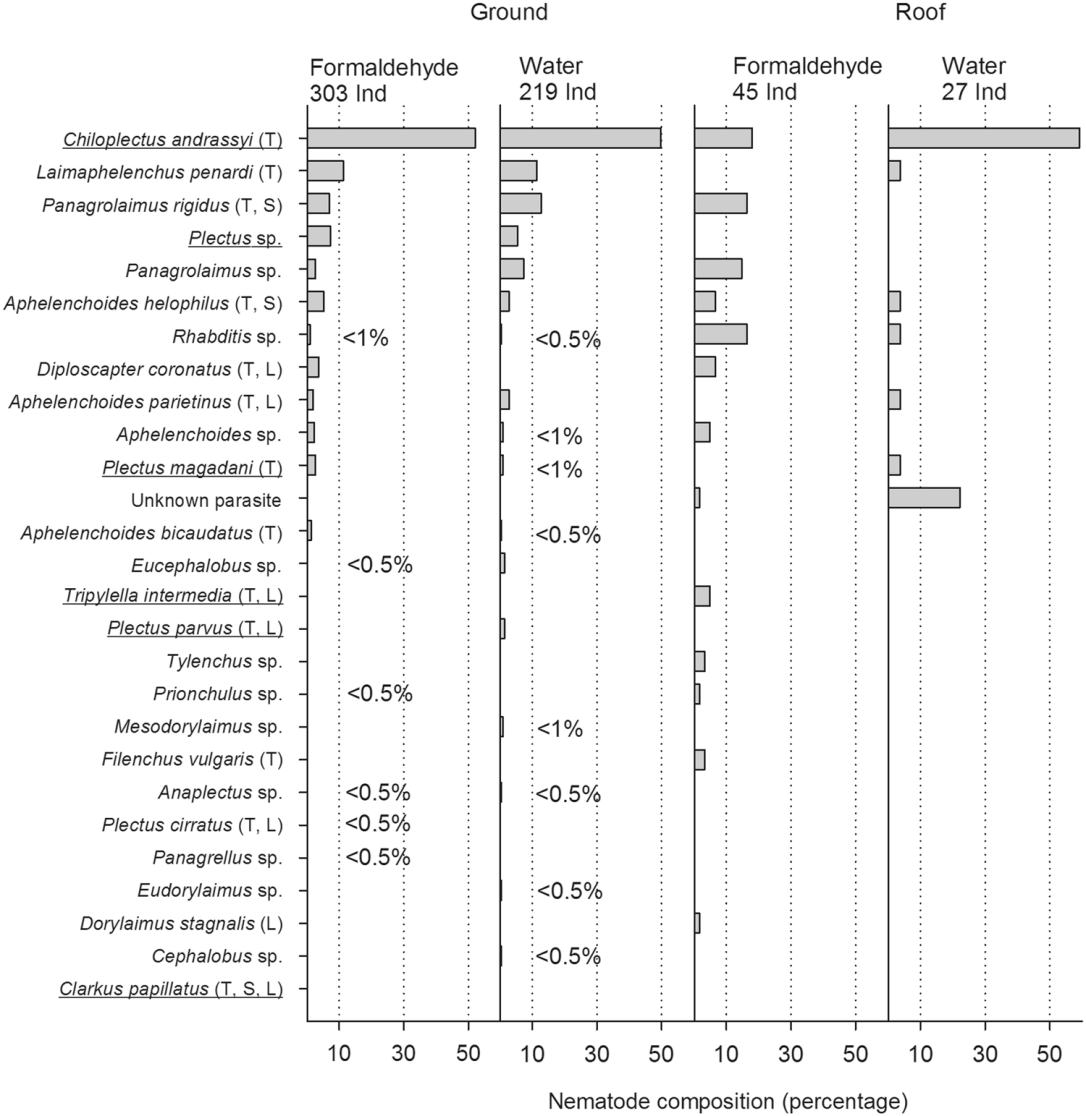
Percentage composition of nematode species from funnels filled with formaldehyde or water placed within a natural environment or on the roof of Bielefeld University. The number of identified individuals is also shown. The letters after the species names indicate their natural occurrence: terrestrial (T), semiaquatic (S), limnic (L). Underlined taxa are those that were also found between stones, organic material, and plants on the roof. The nematode taxa are sorted by frequency.

Due to losses during the fixation process and to damaged individuals, of the 674 nematodes collected in the funnels only 594 could be classified, with 27 species thus identified (Table 2, Fig. 3). In the controls, collected from stones, organic material, and plants on the roof, 108 individuals were identified. More species were found at the natural location (17 in formaldehyde; 17 in water) than on the roof (13 in formaldehyde; 7 in water; 6 in the controls). *Chiloplectus andrassyi* was the most common species in all funnel treatments, accounting for 18.0% of the formaldehyde and 59.3% of the water funnels on the roof and for 52.1% of the formaldehyde and 49.8% of the water funnels on the ground (Fig. 3). Overall, the ten most frequent species made up 74.1–94.4% of the identified nematodes per treatment. The other taxa were mostly single finds, with the exception of an unknown parasite that occurred only on the roof; it represented 22.2% of the nematodes in the water treatment and 1.6% of those in the formaldehyde treatment. Three species (*C*. *andrassyi*, *Plectus* sp. and *Plectus magadoni*) identified on the roof were also collected from stones, organic material, and plants on the rooftop. *Plectus parvus*, *Mesodorylaimus* sp. *Eudorylaimus* sp. and *Cephalobus* sp. were collected only from the water treatments.

**Table 2:** The percentages of size classes and sex distribution of nematodes collected from funnels filled with formaldehyde or water and placed within a natural environment or on the roof of Bielefeld University.

	Ground		Roof		
	Formaldehyde	Water	Formaldehyde	Water	Control
Nematodes found	318	271	57	28	113
Nenatodes identified	303	219	45	27	108
Number of species	17	17	13	7	6
Size classes (%)					
<0,25 mm	7.2	2.6	10.5	3.6	—
0.25–0.5 mm	28.3	34.3	47.4	10.7	—
0.5–0.75 mm	27.7	21.0	29.8	50.0	—
0.75–1 mm	33.3	38.0	10.5	17.9	—
>1 mm	3.5	4.1	1.8	17.9	—
Sex distribution (%)					
juveniles	56.1	61.2	73.8	70.4	80.6
adults	43.9	38.8	26.2	29.6	19.4
adult males	2.6	1.4	6.6	0.0	0.0
adult females	41.3	37.4	19.7	29.6	19.4
gravid females	2.3	4.6	0.0	3.7	0.9

Data from the control, nematodes collected from stones, organic material, or plants on the roof, are also provided. Nematode numbers in the different replicates and from the different sampling dates were summed for each treatment before further calculation. The percentage of size classes is based on the number of collected nematodes, and the sex distribution on the number of identified nematodes.

The largest nematode had a body length of 1.5 mm and was collected from a formaldehyde funnel placed in the natural environment. In general, 63.6% of the nematodes from the ground and 87.7% of those from the roof were <1 mm in size (Table 2).

In all treatments, the share of juvenile nematodes was the largest, comprising at least 56.1% of the collected nematodes (Table 2). Among the samples collected on the roof juveniles made up the largest share (70.4% in water and 73.8% in formaldehyde) whereas at the natural location their share was lower (61.2% in water and 56.1% in formaldehyde). Males (<6.6%) as well as gravid females (<4.6%) were underrepresented in all treatments and were only partly found in samples from the roof.

### Meteorological impact

GLMs were calculated only for nematodes, mites, and thrips as the other taxa (rotifers, tardigrades and collembola) occurred only occasionally in the formaldehyde funnels such that statistical analysis of their abundance data was not possible. The GLMs that, according to the lowest AIC_c_ value and likelihood ratio tests, best fitted the count data of nematodes, mites, and thrips are summarized in Table 3 (for model selection see Table S1). In the interpretations of the estimated contrasts, a logarithmic link function was used; thus, an additive change in the predictor had multiple effects on the response. The model revealed that the number of nematodes in the funnels increased significantly with increasing relative humidity. It also predicted a significantly higher input of nematodes at high wind speeds, whereas this predictor was a less reliable estimate for nematode numbers because of the standard errors (Table 3). The model that predicted the abundance of mites indicated that increasing numbers were significantly related to higher temperatures. The number of thrips in the samples decreased significantly with increasing humidity.

**Table 3:** A generalized linear model using the negative binomial family.

Taxon	Coefficient	Estimate	Standard error	Z value	p
Nematodes	Intercept	−8.95	2.59	−3.46	<**0.001**
	Humidity	0.12	0.03	4.13	<**0.001**
	Wind speed	0.23	0.10	2.28	**0.023**
Mites	Intercept	−0.74	0.49	−1.50	0.133
	Temperature	0.17	0.04	4.62	<**0.001**
Thrips	Intercept	18.96	3.74	5.06	<**0.001**
	Humidity	−0.22	0.05	−4.62	<**0.001**

The response variables were the organism counts in the funnels with formaldehyde (pooled number from the roof and ground samples) and the possible predictors were temperature, humidity, wind speed, and precipitation. The presented models are those that best fit the data during the model selection process (see Table S1).

## Discussion

Our study is one of the first to provide clear information on the number of immigrating organisms caused by wind dispersal. The very large dispersion of nematodes in the complete absence of propagules, including anhydrobiotic stages, was unexpected.

### Components of the aeroplankton

The results indicated that in Central Europe several metazoan taxa are transported by wind and that their dispersal is subject to a strong seasonal dynamic. Of the six groups (mites, tardigrades, collembolans, nematodes, rotifers, and thrips) examined in this study of aeroplankton, nematodes dominated, with dispersal rates as high as 3021 nematodes m^−2^ in 4 weeks. This value reflected the high abundances of nematodes in other habitats and the strong likelihood of wind dispersal, in line with hypothesis H1.

For organisms of all six taxa, the mean dispersal rates were higher on the ground, i.e., closer to potential source habitats, than on the roof, where dispersed organisms were deposited in funnels placed 35 m above ground. This result clearly confirmed our second hypothesis (H2.1), demonstrating both a dispersal limitation and sorting processes for wind-drifted taxa, as shown by Caceres and Soluk^10^ and by Jenkins^12^. It also demonstrated more efficient local wind dispersal within environments linked to several habitat types. The exception was thrips, whose input of individuals was almost as high on the roof as on the ground (Fig. 2), suggesting the higher potential of these organisms for long-distance transport. As this group was the only one of the six taxa that, with its wings, was at least partly able to actively reinforce dispersal^38^, this was a plausible result. However, also for the other groups, regional transport is to some extent possible, as shown by the organisms that over a period of 4 weeks entered the funnels on the roof with a dispersal rate from 2 (tardigrades) to 125 (nematodes) Ind. m^−2^.

The amount and type of wind-dispersed organisms collected in our experiment were substantially different from those reported in other experiments: In a South African study, 950 copepods, 203 cladoceres, and 45 ostracodes were captured within 1 month by windsocks with a surface area of 1 m^2^ ^17^ whereas these groups were not present in any of our funnels. In that same study, 18 mites m^−2^ were detected, which is only a fifth to a tenth (roof and ground, respectively) of the dispersal rates of this group in our study. The dispersal rates of tardigrades and rotifers collected from Antarctica^8^ were also consistently lower (<100 Ind. m^−2^ per year) than those measured in the present work for nematodes (>10,000 Ind. m^−2^ per year), tardigrades (540 Ind. m^−2^ per year), and rotifers (480 Ind. m^−2^ per year) on the ground.

In contrast to our high nematode dispersal rates, those in previous studies were up to 600× lower: Thus, Nkem *et al*.^8^ detected only single nematodes (<5) m^−2^ in Antarctica within one year. Vanschoenwinkel *et al*.^17^ caught aeroplankton by windsocks placed for 1 month on an isolated mountain top in South Africa. Only propagules >100 µm were considered for this study. Based on the data, approximately 14 nematodes were sampled per m^2^. A longer testing period presumably would have led to higher values.

However, because of the substantially different environments (Antarctica, South Africa), a direct comparison of the results of this and previous studies is difficult.

Moreover, the different sampling methods used may have significantly affected the number of collected organisms^8^. In the study of Vanschoenwinkel *et al*.^39^, the composition of aeroplankton captured at the same site and time by windsocks vs. sticky traps strongly varied. It is also likely that most of the other studies considered the incoming organisms after they had interacted with the new habitat, whereas in our formaldehyde-filled funnels all organisms died immediately after their arrival. These two different approaches lead to different measures of dispersal rates. However, we are confident that our method realistically reflects the input of wind-dispersed organisms in a given habitat within the temperate zone.

Nematode dispersal was also considered strictly on a species level. Twenty-seven nematode species entered the experimental vessels, 21 in the natural environment and 15 on the roof, in accordance with H2.2, which predicted higher species richness in funnels with higher proximity to source habitats. Only three of the ten most frequent species on the roof were also found in the samples from the rooftop (controls); thus, the remaining two species most certainly drifted onto the roof by wind, implying their long-distance transport, as described by Carroll and Viglierchio^14^.

Previous studies reported three wind-drifted nematode species from Antarctica^8^, at least 14 species from Senegal^40^, and 10 genera from California^41^. At the species level none of the species in this study overlapped with any of those from other locations. However, many near-relative taxa are dispersed by wind, including those of the most common species of the present study. Rhabditida (in our study: *Panagrolaimus rigidus*, *Panagrolaimus* sp., *Rhabditis* sp., *Diploscapter coronatus*, *Eucephalobus* sp., *Panagrellus* sp., and *Cephalobus* sp.) and Dorylaimida (in our study: *Mesodarylaimus* sp., *Eudorylaimus* sp., and *Dorylaimus stagnalis*) are also frequently found in Senegal, California, and the Antarctic. Members of the genus *Aphelechoides* (in our study: *Aphelenchoides helophilus*, *Aphelenchoides parietinus*, *Aphelenchoides* sp., *Aphelenchoides bicaudatus*) were among the aeroplankton reported from California, while members of the Plectina (in our study: *Chiloplectus andrassyi*, *Plectus* sp., *Plectus magadani*, *Plectus parvus*, *Anaplectus* sp., and *Plectus cirratus*) were collected from the Antarctic. Thus, worldwide, certain taxonomic groups seem to be typically dispersed by wind.

The presence of one parasitic species indicated that nematodes are transported by larger organisms. This species was always collected from funnels containing (drowned) adult dipterans. However, we found no correlation between the numbers of insects and the parasite. Indeed, in the natural environment nematodes reached their highest number in winter, when no insects were collected from the funnels.

*Dorylaimus stagnalis* was the only species whose distribution is restricted to freshwater habitats. All of the other nematodes identified to the species level are mainly terrestrial^28–30^, but some are also found in semiaquatic or aquatic environments. Specifically, the main sources of the collected species were forest soils, leave litter, decomposed organic matter, moss, tree bark, plants, and phytotelmata^11,27,31,42^. Vanschoenwinkel *et al*.^18^ pointed out that the dispersal rates of aquatic invertebrates already drop off at 10–20 m distance to the nearest source pond, which might explain why so few freshwater species were collected in our study. Interestingly, other aquatic invertebrates, including ostracodes, copepods, and cladoceres were also not captured in the funnels (see above).

Also, molecular studies found evidence that dispersal and therefore the gene flow between populations may be more limited than originally thought. For example, Ristau *et al*.^43^ found genetic differentiation of a freshwater nematode species already in neighboring lakes. Also, the study of Suatoni *et al*.^44^ indicated dispersal limitations resulting in the presence of cryptic species of a rotifer species which was previously thought to have a broad geographic distribution and therefore high passive dispersal rates.

### Influence of meteorological factors

For the three tested groups, nematodes, mites and thrips, different meteorological factors were evaluated for their significance as predictors of organismal dispersal rates within the model selection process. While, in general, meteorological parameters were significantly related to dispersal, hypothesis 3 (H3) was not confirmed, as neither dry conditions nor wind speed generally supported the input of all taxa. For mites the highest rate of dispersal occurred in summer (May–October), indicating the significant influenced of temperature on this process. A previous study showed that the density of mites in soil is strongly temperature restricted^45^. In another^46^, mites were present in abundance in the upper layers (0–2.5 cm) of soil ecosystems during the summer but moved deeper into the soil in winter. Thus, in summer more individuals would be vulnerable to wind erosion. Similarly, the dispersal of thrips was also restricted to the warmer months (May–October), consistent with their active phase occurring solely during the warmer months^47^. Although the model selection process identified humidity as the more crucial factor, temperature and humidity are inversely correlated (Pearson’s correlation: *r* = −0.56, *p* = 0.002) such that their individual influences are difficult to distinguish. However, in contrast to the other two groups, the dispersal rates of nematodes were highest in winter (December–March). Our study showed the importance of wind speed for nematode transport and supports the observation that only when the wind energy exceeds a specific threshold organisms can be lifted from the ground^14^. Accordingly, at higher wind speeds more nematodes were deposited in the funnels. Wind speed also regulates the distance of the transport.

Our results also identified moisture as a crucial factor in the wind dispersal of nematodes, as the highest dispersal rates were measured during periods of high humidity. Although it is widely assumed that small organisms are dispersed during dry periods^5^, most of the organisms collected in this study were those adapted to terrestrial environments, including soils. In forest soils, nematode abundances are highest during periods of high moisture^48^, which implies an increased potential for the erosion of these organisms.

### Dispersal stages of nematodes

In terms of the sexual distribution, our results did not differ from those of previous studies of nematode communities in soil and freshwater habitats. However, there was a slight shift towards more juveniles^31,49,50^, mostly those with a body length <0.75 mm. As reported by Nkem *et al*.^8^ and by Carroll and Vigliechio^14^, especially juvenile nematodes can be drifted over long distances. Consistent with this finding, in our study there was a higher ratio of small juvenile nematodes on the roof than on the ground. Their longer transport distance lent support for H4.

Contradicting H5, however, was the observation that all nematodes collected from the formaldehyde treatments were dispersed in an active (living) condition, and only four species (*Plectus parvus*, *Mesodorylaimus* sp., *Eudorylaimus* sp. and *Cephalobus* sp.) were present solely in the water-filled funnels. These may have entered the funnels as propagules and subsequently developed into an active life stage. However, there were few such individuals (<1.5% of the total nematodes) and all other nematode species were found in both treatments, indicating the viability of incoming individuals.

For rotifers and tardigrades in this study, the input of propagules cannot be excluded. Like nematodes, these two groups of organisms can survive frost, heat, and desiccation for months and even years by adopting a state of anhydrobiosis^24,26^ (illustrated by^40,49^). Nematode genera able to outlast unfavorable environmental conditions by anhydrobiosis include those also detected in our study: *Aphelenchoides*, *Panagrellus*, *Plectus*, and *Tylechus*^24,26,51^. The factors that are crucial for a successful transition to anhydrobiosis and a subsequent re-emergence are temperature and moisture^15,51^, with the chances of survival increasing under conditions of slowly rising temperatures, high humidity, and therefore slow drying. Among these factors, our investigation showed that humidity is crucial for nematode dispersal. Previous studies identifying nematodes as part of the aeroplankton were mainly conducted in extreme habitats with harsh living conditions. Nkem *et al*.^8^ sampled wind-dispersed nematodes from Antarctica and found mainly “inactive” individuals. Baujard and Martiny^40^ as well as Vanschoenwinkel *et al*.^17,18^ documented similar forms in Africa during the dry season. By contrast, in their study in California under less harsh conditions, Vigliercho and Schmitt^41^ found living individuals. Thus, in temperate and more humid environments anhydrobiosis may not be necessary for wind dispersal, a conclusion supported by our investigation.

## Conclusion

Our results well demonstrated the wind dispersal of several small metazoans and that their dispersal rates were related to seasonal or/and meteorological influences. A relationship between proximity to the source habitat (forest) and the number of drifted individuals and species was also determined. Based on the organisms deposited in funnels placed on the roof of a ten-story building, we concluded, that even transport on a larger scale is possible.

The wind dispersal of many organisms has been underestimated; this is especially true for nematodes, an ecologically essential group. In temperate zones, the annual immigration of nematodes of numerous species may exceed 10,000 per m^2^, which is much higher than measured for other wind-drifted taxa. Contrary to common assumptions, we demonstrated that neither propagules nor dry environmental conditions are a requirement for nematode dispersal by wind. Moreover, as the incoming nematodes were viable, the wind dispersal of nematodes enables gene flow between populations in very remote habitats and increases both the diversity and the stability of nematode populations. The colonization efficiency of nematodes with respect to other factors, such as landscape fragmentation, was beyond the scope of our study and should be the focus of further investigations.

## Electronic supplementary material

Supplementary Information
