# Supplementary material for: The extent of wind-mediated dispersal of small metazoans, focusing nematodes
Source: Sci Rep. 2018 May 1;8:6814. doi: 10.1038/s41598-018-24747-8 (PMC5931521; doi:10.1038/s41598-018-24747-8)
Supplement: Supplementary file 1 — Supplementary Information [file 41598_2018_24747_MOESM1_ESM.doc]

**Supplementary Information**

**The extent of wind-mediated dispersal of small metazoans, focusing nematodes**

Christoph Ptatscheck*,

christoph.ptatscheck@uni-bielefeld.de

Animal Ecology, Bielefeld University, Konsequenz 45, 33615, Bielefeld, Germany

+49 521 106 2705

Birgit Gansfort,

birgit.gansfort@uni-bielefeld.de

Animal Ecology, Bielefeld University, Konsequenz 45, 33615, Bielefeld, Germany

Walter Traunspurger

traunspurger@uni-bielefeld.de

Animal Ecology, Bielefeld University, Konsequenz 45, 33615, Bielefeld, German

**Table S1**: Model selection results for generalized linear models using the negative binomial family. The response variable were the summarized organism counts in the formaldehyde funnels, the possible predictors were: mean temperature, mean wind speed, mean precipitation and mean humidity during the sample intervals. In the likelihood ratio test, the “best” parsimonious model (in the first line of each section) was tested against each possible model including one additional predictor (forward selection); p-values < 0.05 and the model with the lowest (second order) Akaike information criterion (AICc) value for each taxon are shown in bold.

| Taxon | Formula | Df | AIC | AICc | Null-  Deviance | Residual-  Deviance | 2 x  log-lik | Chi df | LR stat | p |
| --- | --- | --- | --- | --- | --- | --- | --- | --- | --- | --- |
| Nematoda | 1 | 27 | 206.33 | 206.48 | 32.17 | 32.17 | -202.33 |  |  |  |
|  | 1 + temperature | 26 | 202.58 | 203.06 | 38.12 | 31.79 | -196.58 | 1 | 5.75 | **0.016** |
|  | 1 + wind speed | 26 | 205.03 | 205.51 | 35.44 | 31.96 | -199.03 | 1 | 3.29 | 0.070 |
|  | 1 + percipitation | 26 | 205.21 | 205.69 | 35.14 | 31.69 | -199.21 | 1 | 3.11 | 0.078 |
|  | 1 + humidity | 26 | 199.57 | 200.05 | 41.33 | 31.27 | -193.57 | 1 | 8.75 | **0.003** |
|  | 1 + humidity | 26 | 199.57 | 200.05 | 41.33 | 31.27 | -193.57 |  |  |  |
|  | 1 + humidity + temperatur | 25 | 200.77 | 201.77 | 42.41 | 31.26 | -192.77 | 1 | 0.80 | 0.371 |
|  | **1 + humidity + wind speed** | **25** | **194.93** | **195.93** | **51.65** | **31.28** | **-186.93** | **1** | **6.64** | **0.010** |
|  | 1 + humidity + percipitation | 25 | 198.95 | 199.95 | 44.85 | 31.12 | -190.95 | 1 | 2.62 | 0.105 |
| Acari | 1 | 27 | 142.72 | 142.87 | 29.95 | 29.95 | -138.72 |  |  |  |
|  | **1 + temperature** | **26** | **130.02** | **130.50** | **52.17** | **31.02** | **-124.02** | **1** | **14.70** | **<0.001** |
|  | 1 + wind speed | 26 | 143.58 | 144.06 | 31.07 | 29.91 | -137.58 | 1 | 1.14 | 0.287 |
|  | 1 + percipitation | 26 | 143.20 | 143.68 | 31.75 | 30.17 | -137.20 | 1 | 1.52 | 0.218 |
|  | 1 + humidity | 26 | 140.49 | 140.97 | 33.92 | 29.40 | -134.49 | 1 | 4.23 | **0.040** |
|  | 1 + temperature | 26 | 130.02 | 130.50 | 52.17 | 31.02 | -124.02 |  |  |  |
|  | 1 + temperature + humidity | 25 | 130.12 | 131.12 | 52.13 | 29.10 | -122.12 | 1 | 1.90 | 0.168 |
|  | 1 + temperature + wind speed | 25 | 131.28 | 132.28 | 53.41 | 30.91 | -123.28 | 1 | 0.75 | 0.388 |
|  | 1 + temperature + percipitation | 25 | 131.64 | 132.64 | 54.29 | 31.71 | -123.64 | 1 | 0.39 | 0.534 |
| Thysanoptera | 1 | 27 | 155.92 | 156.07 | 29.35 | 29.35 | -151.92 |  |  |  |
|  | 1 + temperature | 26 | 157.79 | 158.27 | 29.48 | 29.35 | -151.79 | 1.00 | 0.13 | 0.716 |
|  | 1 + wind speed | 26 | 157.40 | 157.88 | 29.85 | 29.33 | -151.41 | 1.00 | 0.51 | 0.473 |
|  | 1 + percipitation | 26 | 157.48 | 157.96 | 29.80 | 29.35 | -151.48 | 1.00 | 0.44 | 0.505 |
|  | **1 + humidity** | **26** | **148.44** | **148.92** | **39.84** | **28.53** | **-142.44** | **1.00** | **9.48** | **0.002** |
|  | 1 + humidity | 26 | 148.44 | 148.92 | 39.84 | 28.53 |  |  |  |  |
|  | 1 + temperature + humidity | 25 | 150.20 | 151.20 | 39.66 | 28.17 | -142.20 | 1.00 | 0.24 | 0.624 |
|  | 1 + temperature + wind speed | 25 | 150.08 | 151.08 | 39.74 | 28.10 | -142.08 | 1.00 | 0.36 | 0.549 |
|  | 1 + temperature + percipitation | 25 | 150.22 | 151.22 | 40.17 | 28.53 | -142.22 | 1.00 | 0.22 | 0.638 |
